# Temporal expression classes and functions of vaccinia virus and mpox (monkeypox) virus genes

**DOI:** 10.1128/mbio.03809-24

**Published:** 2025-03-20

**Authors:** Yining Deng, Santiago Navarro-Forero, Zhilong Yang

**Affiliations:** 1 Department of Veterinary Pathobiology, College of Veterinary Medicine & Biomedical Sciences, Texas A&M University 199063 , College Station, Texas, USA; The Ohio State University, Columbus, Ohio, USA

**Keywords:** mpox virus, monkeypox, vaccinia virus, poxvirus, gene expression, function

## Abstract

Poxviruses comprise pathogens that are highly pathogenic to humans and animals, causing diseases such as smallpox and mpox (formerly monkeypox). The family also contains members developed as vaccine vectors and oncolytic agents to fight other diseases. Vaccinia virus is the prototype poxvirus and the vaccine used to eradicate smallpox. Poxvirus genes follow a cascade temporal expression pattern, categorized into early, intermediate, and late stages using distinct transcription factors. This review comprehensively summarized the temporal expression classification of over 200 vaccinia virus genes. The relationships between expression classes and functions, as well as different branches of immune responses, were discussed. Based on the vaccinia virus orthologs, we classified the temporal expression classes of all the mpox virus genes, including a few that were not previously annotated with orthologs in vaccinia viruses. Additionally, we reviewed the functions of all vaccinia virus genes based on the up-to-date published papers. This review provides a readily usable resource for researchers working on poxvirus biology, medical countermeasures, and poxvirus utility development.

## AN OVERVIEW OF TEMPORAL CASCADE EXPRESSION OF POXVIRUS GENES

Many members of poxviruses are significant pathogens causing high-consequence diseases in humans or animals. The orthopoxviral genus is particularly significant as it includes members closely related to human health among the over 20 genera of the poxvirus family. Some orthopoxviruses, such as variola virus that causes smallpox and mpox virus (or monkeypox virus, MPXV) that causes mpox, pose severe threats to global public health. Meanwhile, some other poxvirus members, such as vaccinia virus (VACV), are effective vaccines that were used to eradicate smallpox and are developed as vaccine vectors and oncolytic virotherapy.

Poxviruses harbor large double-stranded DNA (dsDNA) genomes ranging from 130 kbp to over 300 kbp, encoding a sizable number of open reading frames (ORFs). VACV, the prototype poxvirus, has a genome of 197 kbp with over 200 annotated ORFs. Early work demonstrated that poxvirus genes are expressed in a cascade manner with early, intermediate, and late stages, each with their own specific transcription factors, and are regulated by stage-specific transcription factors. The early genes start to be transcribed immediately after viral entry into the cells in the cores before viral DNA replication using virally encoded early transcription factors, DNA-dependent RNA polymerases, and other RNA processing factors incorporated into the viral core. The early gene products include DNA polymerase, other DNA replication factors, and intermediate transcription factors. The accumulated viral DNA and intermediate transcription factors allow the initiation of transcription of intermediate viral genes, which encode proteins including late transcription factors. The synthesis of intermediate viral transcription factors prompts the transcription of viral late genes, whose products include viral early transcription factors. The early transcription factors, viral RNA polymerase, and processing factors are assembled in the virions, allowing immediate transcription of early genes in a new round of infection. Numerous pioneering studies characterized the cascade transcription program and the molecular mechanism of VACV RNA polymerase action (1–3). We apologize that we are not able to cite all these publications here. A few detailed reviews on vaccinia virus transcription system, RNA polymerase, and mechanisms of action can be found elsewhere (1–3). Rather, this review focuses on the classification of individual poxvirus genes in the three cascade stages and their functions, serving as a resource for the community studying poxviruses, especially VACV and MPXV.

## CLASSIFICATION OF THE TEMPORAL EXPRESSION OF VACV GENES

While a precise classification of the temporal expression of all individual poxvirus genes is critical to understanding many aspects of viral replication mechanism and host–virus interaction, the task had been exceptionally challenging due to i) the closely spaced, sometimes overlapped ORFs, and ii) extensive readthroughs of many transcripts, especially of intermediate and late transcripts that are adjacent to other ORFs. Considerable efforts using classic molecular biology approaches during earlier era had classified a number of VACV genes into early, intermediate, and late stages. However, these methods are only practical for classifying a limited number of genes. The effort required for over 200 genes would be tremendous. The differentiation of intermediate and late genes is particularly challenging based on the timing of expression as their expression timings are very close, there are extensive RNA readthroughs, and no highly stringent genetic or chemical tools are currently available to distinguish between them. Several earlier attempts using microarray-based RNA profiling yielded considerable misclassifications due to insufficient resolution and background signal noise (4–6).

RNA-seq with digital quantification at nucleotide resolution and minimal background noise, combined with cytarabine (AraC) treatment to halt VACV replication before the viral DNA replication stage, allowed the identification of 118 early genes that are expressed before viral DNA replication (7). However, the intermediate and late genes are indistinguishable using the RNA-seq data due to extensive readthroughs. Based on protein expression of individual genes from plasmids in the presence or absence of late transcription factors in VACV-infected cells, Yang *et al*. characterized that 53 VACV genes initiate their expression at the intermediate stage and 38 genes initiate their expression at the late stage (8). Some genes are expressed at multistages due to hybrid promoters containing more than one promoter motif (9), which has not been completely characterized. Based on the classifications, we used the VACV Western Reverse (WR) strain as a model and summarized the temporal expression cascade of all genes based on their transcription onsets (Table 1). Although the poxvirus genome is linear, its structure is unique because the terminal regions of the genome are inverted terminal repeats (ITRs). These ITRs contain identical sequences at the 5' and 3' ends, effectively creating an overlapping region. As a result, the same genes located at the ends of the genome are given two different names—one for the left end and another for the right end. This naming convention reflects the duplication of terminal genes within the ITRs, which are crucial for genome stability, replication, and interactions with the host.

**TABLE 1:** MPXV genes and their homolog in VACV WR

Gene name					Expression classes^*c*^	AA identity (%)	Functions^*d*^	Function reference(s)
OPG^*a*^	VACV WR	VACV-COP orthologs^*b*^	MPXV orthologs (based on MPXV-USA_2022_MA001)^*b*^					
001	01/218	C23L/B29R	001–001/190	J1L	E	84.71	Encoding a 7.5 KDa protein (the GenBank annotation is inaccurate). CC-chemokine binding chemokine binding protein, secreted protein, non-essential in cell culture	(10, 11)
002	02/217	Pseudogene (L)	002/189	J2L	E	91.11	Gene fragment, TNFα-receptor-like; unknown function	
002	03/216	No ortholog			NE		Gene fragment, not translated	(11)
002	04/215	C22L/B28R	002/189	J2L	E	83.52	Gene fragment, TNF receptor (CrmB), secreted TNF-binding protein, TNFα-receptor-like;	(12, 13)
003	005/214	Pseudogene (L)	003/188	J3L	E	93.88	Gene fragment, ankyrin-like, unknown function	
003	006/213	C21L/B27R	003/188	J3L	E	100	Gene fragment, ankyrin-like, preventing antibody-dependent complement-enhanced neutralization of infectivity and contributing to virulence	(14)
003	007/212	C20L/B26R	003/188	J3L	E	80.81	Gene fragment, unknown function	
003	008/211	C19L/B25R	003/188	J3L	L	87.50	Gene fragment, ankyrin-like, unknown function	
019	009/210	C11R	006	D3R	E	93.53	Secreted EGF-like growth factor, modulating host metabolism, promoting virulence, and facilitating cell motility and virus spread	(15–21)
020	010/209	C10L	007	D4L	E	94.59	IL-1 receptor antagonist, immunosuppressive activity, host defense modulator, and virulence factors, reprogramming cellular energy metabolism (named C16 in some studies)	(22–24)
021	011/208	No ortholog	008	D5R	E	95.11	Zinc finger-like, apoptosis inhibition, induced by UV irradiation and virosome localization	(15)
021	012/207	No ortholog (L)	008	D5R	E	91.23	Zinc finger-like protein	
022	013	No ortholog	009	D6L	E	82.54	Soluble secreted IL-18 binding protein, immune evasion, and virulence (named C12 in the referenced study)	(25)
023	014	No ortholog (L)	010	D7L	E	94.54	Ankyrin-like protein	
023	015	No ortholog (L)	010	D7L	E	91.91	Ankyrin-like protein	
023	016	No ortholog (L)	010	D7L	E	98.39	Ankyrin-like protein	
023	017	No ortholog (L)	010	D7L	E	95.65	Ankyrin-like protein	
024	018	No ortholog (L)	011	D8L	E	74.07	Binding to the SH2 domain of STAT1, type I IFN inhibitor, host range factor, antagonist of host restriction factors	(26)
025	019	C9L	012	D9L	E	85.33	Ankyrin-like, antagonist of type-I IFN	(27)
026	020	C8L			L		Unknown function	
027	021	C7L	013	D10L	E	98.00	Type I IFN inhibitor, host range factor, antagonist of host restriction factors, SAMD9, and SAMD9L	(28–33)
029	022	C6L	014	D11L	E	91.72	Bcl-2-like protein, IFN-β inhibitor, host-range, virulence factor, binds TBK-1 adapter proteins, and inhibits activation of IRF3 and IRF7	(34, 35)
030	023	C5L	015	D12L	E	94.30	BTB domain of kelch-like protein, associated with cullin-3-based ligase complexes, uncoating, and DNA replication factor	(36)
031	024	C4L	016	D13L	E	94.62	Inhibiting NF-κB activation and promoting virus virulence	(37)
032	025	C3L		D14L^*e*^	L	95.61	Secreted complement binding [C3b/C4b]; host defense modulator	(38, 39)
033	026	C2L	017	D18L*^f^*	E	96.20	POZ/BTB kelch domain protein, affecting calcium-independent adhesion to the extracellular matrix and inflammation in a murine intradermal model, inhibiting NF-κB activation	(40–42)
034	027	C1L	018	D19L	E	94.39	Modulating the host’s immune response through inflammasomes, putative TLR signaling inhibitor	(43, 44)
035	028	N1L	019	P1L	E	89.74	Anti-apoptotic, Bcl-2-like protein, host defense modulator, and inhibits NF-κB and IRF3 activation	(45–48)
036	029	N2L	020	P2L	E	92.00	α-amanitin target protein, nuclear IRF3 inhibitor, promoting virulence	(49)
037	030	M1L	021	O1L	E	96.84	Ankyrin-like protein and apoptosis inhibitor	(50, 51)
038	031	M2L	022	O2L	E	97.27	NF-κB inhibitor, altering host-mediated immune responses, uncoating, and DNA replication factor	(36, 52, 53)
039	032	K1L	023	C1L	E	95.77	Ankyrin-like protein, NF-κB inhibitor, needed for viral replication and is capable of complementing for C7L function, antagonist of host restriction factors, SAMD9, and SAMD9L	(30–32, 54–56)
040	033	K2L	024	C2L	I	91.96	SPI-3, inhibiting infected cells to fuse serine protease, prevents syncytia, interacting with A56 to form fusion regulatory complex, and inhibiting syncytia	(54, 57–61)
041	034	K3L	025	C3L	E	95.24	IFN resistance, homolog of eIF2α, inhibiting PKR. MPXV ortholog lost the essential PKR-interacting motif due to the premature stop codon inside.	(62–64)
042	035	K4L	026	C4L	I	97.41	Phospholipase-D-like protein, nicking/joining enzyme	(65, 66)
043	036	Pseudogene (L)	028	C6R	E	100	Gene fragment, putative monoglyceride lipase, unknown function	(54, 64, 67)
043	037	K5L	027	C5L	E	78.63	Gene fragment, putative monoglyceride lipase, unknown function	(54, 64, 67)
043	038	K6L	028	C6R	E	92.00	Gene fragment, putative monoglyceride lipase, unknown function	(64)
044	039	K7R	028	C6R	E	94.63	Host immune response repressor, inhibiting PKR-mediated induction of IFN-β, interacting with DDX3, and promoting histone acetylation	(68, 69)
045	040	F1L	029	C7L	E	85.90	Caspase-9 (apoptosis) inhibitor (mitochondrial-associated), blocking ribotoxic stress response	(70–73)
046	041	F2L	030	C8L	E	97.96	dUTPase deoxy uridine triphosphatase, involved in nucleotide metabolism	(74, 75)
047	042	F3L	031	C9L	E	96.66	Kelch-like, virulence, inhibiting NF-κB activation	(42, 76)
048	043	F4L	032	C10L	E	98.75	Ribonucleotide reductase, small subunit	(77, 78)
049	044	F5L	033	C11L	E	92.57	Membrane protein, required for normal plaque morphology	(79, 80)
050	045	F6L	034	C12L	E	96.00	Unknown function	
051	046	F7L	035	C13L	E	93.42	Unknown function	
052	047	F8L	036	C14L	E	96.92	Cytoplasmic protein, protein with iActA-like proline repeats, not required for actin tail formation	(81)
053	048	F9L	037	C15L	L	98.58	Part of the vaccinia virus entry–fusion complex, S-S bond formation pathway protein substrate	(82, 83)
054	049	F10L	038	C16L	L	99.32	Essential Ser/Thr kinase, required for morphogenesis	(84–87)
055	050	F11L	039	C17L	E	95.20	RhoA signaling inhibitor, virus release protein, stimulating microtubule dynamics, facilitating cell detachment, and promoting migration	(88, 89)
056	051	F12L	040	C18L	E	96.86	Enveloped virion maturation protein, actin tail formation, involved in plaque and enveloped virion formation, and association with intracellular enveloped virions	(90–92)
057	052	F13L	041	C19L	I	99.19	Palmitoylated enveloped virion membrane glycoprotein, phospholipase D-like, major envelope antigen of enveloped virion wrapping, phospholipase motif, required for enveloped virion formation, and target of tecovirimat	(93, 94)
058	053	F14L	042	C20L	E	98.63	Inhibiting NF-κB activation and promoting virulence	(95)
059	053.5	F14.5L	043	C20.5L	E	93.88	IMV protein, important for calcium-independent cell adhesion and virulence in mice	(96)
060	054	F15L	044	C21L	E	98.10	Unknown function	
061	055	F16L	045	C22L	E	96.10	Predicted inactive serine recombinase targets to nucleoli	(97)
062	056	F17R	046	C23R	L	97.03	DNA-binding phosphoprotein (VP11), core protein, may be an mTOR antagonist, counteracting mitochondrially orchestrated antiviral responses	(98–101)
063	057	E1L	047	F1L	E	98.96	Poly (A) polymerase catalytic subunit (VP55), catalytic subunit	(102, 103)
064	058	E2L	048	F2L	E	97.96	Extracellular virion formation, virus spread	(104)
065	059	E3L	049	F3L	E	88.89	IFN resistance/PKR inhibitor (Z-DNA binding), two forms (25 kDa and 19 kDa) of the dsRNA-binding protein MPXV ortholog do not encode the functional Z-DNA binding domain.	(105–107)
066	060	E4L	050	F4L	E	97.68	RNA polymerase 30 kDa subunit RNA (RPO30)	(108)
067	061	E5R	051		E	88.60	Virosome component, inhibitor of DNA sensor cGAS	(109, 110)
068	062	E6R	052	F5R	I	98.94	Virion protein, required for morphogenesis and virion morphogenesis	(111, 112)
069	063	E7R	053	F6R	I	93.98	Soluble myristylated protein has N-myristyltransferase target MGxxxS/T/A	(113)
070	064	E8R	054	F7R	I	98.90	ER-localized membrane protein, virion core protein, required for formation of transcriptionally active virions	(114, 115)
071	065	E9L	055	F8L	E	98.31	DNA polymerase, catalytic subunit	(116)
072	066	E10R	056	F9R	L	95.79	Sulfhydryl oxidase (FAD-linked) protein, disulfide bond-forming enzyme, substrates L1R/F9L, required for morphogenesis, and component of protein disulfide bond formation	(82, 117)
073	067	E11L	057	F10L	I	97.67	Virion core protein, required for virion infectivity	(118)
074	068	O1L	058	Q1L	E	97.00	Membrane protein, viral virulence, sustaining activation of extracellular signal-regulated kinase 1/2, contributing to cytopathic effects (CPE) *in vitro*	(119)
075	069	O2L	059	Q2L	I	98.15	Virion-associated nonessential glutaredoxin; not part of E10R-G4L S-S bond formation pathway	(120)
076	069.5	O3L	59.5	Q3L^*g*^	I	94.29	Component of the vaccinia virus entry/fusion complex	(121)
077	070	I1L	060	I1L	I	99.36	DNA-binding core protein, virosomal protein, essential for virion assembly, and interaction with viral telomeres	(122, 123)
078	071	I2L	061	I2L	L	98.63	Membrane protein with an essential role in viral morphogenesis and entry	(124, 125)
079	072	I3L	062	I3L	E	98.51	Interacting with the subunit of ribonucleotide reductase, ssDNA-binding phosphoprotein	(126, 127)
080	073	I4L	063	I4L	E	99.09	Ribonucleotide reductase, large subunit	(77, 78)
081	074	I5L	064	I5L	I	93.67	Mature virion surface membrane protein, enhancing replication and virulence in mice	(128, 129)
082	075	I6L	065	I6L	I	98.43	Telomere-binding protein, required for morphogenesis	(122, 130)
083	076	I7L	066	I7L	L	98.58	Virion core cysteine protease, similar to DNA topoisomerase II, required for morphogenesis	(131, 132)
084	077	I8R	067	I8R	I	97.49	DNA and RNA helicase, DExH-NPH-II domain, essential for early transcription	(133, 134)
085	078	G1L	068	G1L	L	98.31	Metalloprotease-like, required for formation of infectious virion	(135, 136)
086	079	G3L	069	G2L	L	98.20	Component of the entry/fusion complex component	(137)
087	080	G2R	070	G3R	E	98.64	Putative late transcription elongation factor	(138, 139)
088	081	G4L	071	G4L	I	99.19	Involved in virion-associated glutaredoxin S-S bond formation pathway; thioredoxin-like, required for morphogenesis	(120, 140)
089	082	G5R	072	G5R	E	99.08	FEN1-like nuclease, required for homologous recombination, double-strand break repair, and full-size genome formation.	(141, 142)
090	083	G5.5R	073	G6R	E	100.00	RNA polymerase subunit (RPO7)	(143)
091	084	G6R	074	G7R	L	97.58	NLPc/P60 superfamily protein contributes to virulence in mice but not to replication in cell culture	(144)
092	085	G7L	075	G8L	L	98.92	Virion phosphoprotein, early morphogenesis	(145, 146)
093	086	G8R	076	G9R	I	99.23	Late transcription factor	(147)
094	087	G9R	077	G10R	L	98.24	Entry/fusion complex component, myristyl protein	(113, 148)
095	088	L1R	078	M1R	L	98.40	Mature virion membrane protein, target of neutralizing antibody; S-S bond formation pathway, thiol substrate; required for cell entry and membrane formation	(149, 150)
096	089	L2R	079	M2R	E	96.34	Formation of a crescent membrane, viral membrane assembly proteins (VMAP) recruit endoplasmic reticulum (ER)-derived membranes to form immature virions	(151, 152)
097	090	L3L	080	M3L	L	96.57	Internal virion protein, required for early transcription by cores	(153)
098	091	L4R	081	M4R	I	98.41	ssDNA-/ssRNA-binding protein, involved in early mRNA regulation, stimulates I8R helicase activity	(154–156)
099	092	L5R	082	M5R	L	99.22	Entry/fusion protein	(157, 158)
100	093	J1R	083	L1R	I	97.33	Virion membrane protein, required for morphogenesis	(159)
101	094	J2R	084	L2R	E	97.18	Thymidine kinase	(160)
102	095	J3R	085	L3R	E	98.80	Poly (A) polymerase small subunit (VP39), cap-specific mRNA (nucleoside-O2'-)-methyltransferase, transcription elongation factor	(161, 162)
103	096	J4R	086	L4R	E	100.00	RNA polymerase subunit (RPO22)	(163)
104	097	J5L	087	L5L	L	97.74	MV membrane protein, essential for virus multiplication, component of entry–fusion complex	(157, 164)
105	098	J6R	088	L6R	E	99.07	RNA polymerase subunit (RPO147)	(163)
106	099	H1L	089	H1L	I	98.83	Tyr/Ser phosphatase, IFN-γ inhibitor, required for early transcription, binding, and dephosphorylating STAT1	(165, 166)
107	100	H2R	090	H2R	L	99.47	Mature virion membrane protein, component of the poxvirus multiprotein entry–fusion complex	(167)
108	101	H3L	091	H3L	I	93.83	Mature virion heparin binding surface protein, MV membrane protein, involved in virion maturation, a major target of neutralizing antibodies in humans	(168, 169)
109	102	H4L	092	H4L	L	98.24	RNA polymerase-associated protein (RAP94), virion core, viral early transcriptional factor VETF, conferring early promoter specificity, aiding early-stage transcription preinitiation and termination	(170, 171)
110	103	H5R	093	H5R	E	89.80	Ca2+-binding motif, required for morphogenesis, substrate of B1R kinase, involved in viral DNA replication	(172, 173)
111	104	H6R	094	H6R	L	99.36	DNA topoisomerase, type I topoisomerase, required for morphogenesis	(174, 175)
112	105	H7R	095	H7R	I	97.26	Viral membrane assembly proteins (VMAP), contributing to the formation of crescent membrane precursors of immature virions	(176, 177)
113	106	D1R	096	E1R	E	98.93	mRNA capping enzyme large subunit, transcription termination factor	(178–180)
114	107	D2L	097	E2L	L	97.26	Virion core protein, required for morphogenesis	(181)
115	108	D3R	098	E3R	L	96.20	Virion core protein, required for morphogenesis	(181)
116	109	D4R	099	E4R	E	98.62	Uracil-DNA glycosylase, DNA polymerase processivity factor, interacts with A20 (component of the DNA polymerase processivity factor).	(182–184)
117	110	D5R	100	E5R	E	99.49	NTPase, DNA primase, and nucleic acid-independent nucleoside triphosphatase, essential for DNA replication	(185)
118	111	D6R	101	E6R	I	99.37	Morphogenesis, viral early transcription factor small subunit	(186, 187)
119	112	D7R	102	E7R	E	97.52	RNA polymerase subunit (RPO18)	(188, 189)
120	113	D8L	103	E8L	I	94.41	Carbonic anhydrase, GAG-binding virion membrane protein, mature virion adsorption to cell surface, affects viral entry	(190, 191)
121	114	D9R	104	E9R	E	97.65	mRNA decapping enzyme	(192)
122	115	D10R	105	E10R	I	98.79	mRNA decapping enzyme, promoting viral RNA translation, localizing to mitochondria	(193–195)
123	116	D11L	106	E11L	I	99.05	ATPase, NPH1, DNA-dependent ATPase, and early gene transcription termination factor interact with RAP94	(196, 197)
124	117	D12L	107	E12L	E	98.61	mRNA(guanine-N7-)-methyl-transferase mRNA capping enzyme, small subunit, and transcription initiation factor	(198)
125	118	D13L	108	E13L	I	98.91	Trimeric virion coat protein, needed for the formation of the IMV surface membrane	(199, 200)
126	119	A1L	109	A1L	I	98.67	Viral late transcription factor	(147, 201)
127	120	A2L	110	A2L	I	99.55	Viral late transcription factor	(147, 202)
128	121	A2.5L	111	A3L	L	90.91	S-S bond formation pathway protein, required for morphogenesis	(82, 203)
129	122	A3L	112	A4L	I	99.07	P4b precursor, major virion core protein p4b, membrane associated, required for morphogenesis	(204, 205)
130	123	A4L	113	A5L	E	93.95	39 kDa immunodominant virion core protein, needed for infectious virion formation, 39 kDa virion core protein, required for morphogenesis	(206, 207)
131	124	A5R	114	A6R	E	97.56	DNA-dependent RNA polymerase subunit (RPO19), precursor of RNA polymerase 22 kDa and 21 kDa	(208)
132	125	A6L	115	A7L	I	97.85	Viral membrane assembly proteins (VMAP), core protein, interacting with A21, required for mature virion formation	(209)
133	126	A7L	116	A8L	L	98.45	Viral early transcription factor, large subunit needed for morphogenesis of the virion core	(186)
134	127	A8R	117	A9R	E	98.61	Viral intermediate transcription factor, small subunit	(210)
135	128	A9L	118	A10L	L	88.89	Viral membrane associated, early morphogenesis protein	(211)
136	129	A10L	119	A11L	L	97.08	P4a precursor, major virion core protein, complexes with A4 (p4b), required for morphogenesis	(212)
137	130	A11R	120	A12R	L	99.06	Viral membrane assembly proteins (VMAP), required for morphogenesis	(213)
138	131	A12L	121	A13L	I	96.35	Virion core protein, morphogenesis	(145, 214)
139	132	A13L	122	A14L	L	91.30	Mature virion inner and outer membrane protein, virion maturation, required for morphogenesis	(145, 215)
140	133	A14L	123	A15L	L	100.00	Essential mature virion membrane protein, required for morphogenesis, interacts with A17, F10, and H1 substrate	(216)
141	134	A14.5L	124	A15.5L	L	98.11	Nonessential hydrophobic virion membrane protein; contributing to virus virulence	(217)
142	135	A15L	125	A16L	I	98.94	A component of the seven-protein complex required for the association of membranes and viroplasm to form immature virions	(218)
143	136	A16L	126	A17L	I	97.35	Soluble myristylated protein, component of entry–fusion complex	(157, 219)
144	137	A17L	127	A18L	L	97.55	Mature virion membrane protein, early function in virion morphogenesis	(220)
145	138	A18R	128	A19R	E	95.74	Virion core-associated DNA helicase, DNA-dependent ATPase	(133, 221, 222)
146	139	A19L	129	A20L	I	97.40	Zinc finger-like protein, transformation of spherical immature particles to barrel-shaped infectious virions	(223)
147	140	A21L	130	A21L	L	98.29	Mature virion membrane protein, entry/fusion complex component	(157, 224)
148	141	A20R	131	A22R	E	97.18	Stoichiometric component of the DNA polymerase processivity factor	(225–227)
149	142	A22R	132	A23R	I	97.33	Holliday junction resolvase resolves viral DNA concatemers into the unit length genome	(228)
150	143	A23R	133	A24R	E	98.17	Viral intermediate transcription factors, large subunit	(210)
151	144	A24R	134	A25R	E	99.14	RNA polymerase subunit (RPO132)	(229)
152	145	A25L			NE		Gene fragment, cowpox A-type inclusion protein, MV specific in vaccinia	(230, 231)
152	146		136	A27L	I	36.51	Gene fragment, cowpox A-type inclusion protein	(230, 231)
152	147		135	A26L	I	47.06	Gene fragment, A-type inclusion protein	(230, 231)
152	148		136	A27L	I	95.40	Gene fragment, A-type inclusion protein, MV membrane-associated proteins	(230, 231)
153	149	A26L	137	A28L	L	92.53	Gene fragment, cowpox A-type inclusion protein, p4c protein, complexing with A27, association with A17, binding to the extracellular cellular matrix laminin, fusion suppressor	(232, 233)
154	150	A27L	138	A29L	I	94.55	Mature virion surface membrane fusion protein, binding to cell surface heparan; required for MV wrapping	(145, 232, 234)
155	151	A28L	139	A30L	L	96.58	Component of multiple entry–fusion complex	(157, 235, 236)
156	152	A29L	140	A31L	E	97.70	RNA polymerase subunit (RPO35)	(237)
157	153	A30L	141	A32L	I	94.87	Mature virion protein, core protein, required for morphogenesis	(238, 239)
158	153.5	A30.5L	142		I	87.18	Viral membrane assembly proteins (VMAP), putative transmembrane domain, and colocalized with markers of the endoplasmic reticulum and with L2	(240)
159	154	A31R	143	A33R	E	83.45	Hypothetical protein, putative ATPase	
160	155	A32L	144	A34L	I	98.52	ATPase/DNA packaging protein	(241)
161	156	A33R	145	A35R	E	95.56	Enveloped virion envelope glycoprotein, needed for formation of actin-containing microvilli and cell-to-cell spread of enveloped virion membrane phosphoglycoprotein, C-type lectin-like domain, repulsing superinfecting virions	(242, 243)
162	157	A34R	146	A36R	I	97.02	C-type lectin-like glycoprotein, required for infectivity of enveloped virion, cell-to-cell spread, and formation of actin-containing microvilli	(244)
163	158	A35R	147	A37R	E	97.73	MHC class II antigen presentation inhibitor, intracellular enveloped virion transmembrane phosphoprotein, used in actin tail formation, and helps virion repulsion and rapid spread	(245, 246)
164	159	A36R	148	A38R	E	95.02	Intracellular enveloped virion transmembrane phosphoprotein, actin tail formation, and viral release, repulsing superinfecting virions	(247–251)
165	160	A37R	149	A39R	E	97.72	Unknown function	
166	161	Pseudogene (R)	149.5	A39.5R^*g*^	E	79.37	Unknown function	
167	162	A38L	150	A40L	I	95.29	CD47-like, immunoglobulin-like, integral membrane protein, and regulation of the influx of extracellular Ca2+	(252)
168	163	A39R			L		Gene fragment, semaphorin-like, intact protein proinflammatory in mouse skin lesion model; host defense modulator	(253)
168	164	A39R			I		Gene fragment, semaphorin-like; intact protein proinflammatory in the mouse skin lesion model; host defense modulator	(253)
169	165	A40R			E		C-type lectin-like type-II membrane protein, host defense modulator	(254)
170	166	A41L	151	A41L	E	90.95	Chemokine binding protein, secreted protein reducing the infiltration of inflammatory cells into the infected area secreted glycoprotein	(255, 256)
171	167	A42R	152	A42R	I	97.74	Profilin-like protein, ATI-localized, and trace amount found in mature virions	(257)
172	168	A43R	153	A43R	I	91.84	Type I membrane glycoprotein, enhancing intradermal lesion formation	(258)
173	169	A43.5R	154	A44R	E	98.31	Inhibiting translation imitation, suppressing innate and adaptive immunity, and altering virus virulence	(259)
174	170	A44L	155	A45L	E	98.84	3-β-Hydroxy-Δ5-steroid dehydrogenase, deletion attenuates intradermal lesion in the mouse model; host defense modulator	(260, 261)
175	171	A45R	156	A46R	L	97.60	Inactive Cu-Zn superoxide dismutase-like virion protein	(262)
176	172	A46R	157	A47R	E	95.42	IL-1/TLR signaling inhibitor, suppresses TIR-dependent signal transduction	(263)
177	173	A47L			E		Immunoprevalent protein, homolog of gasdermins, the executioners of pyroptosis.	(264, 265)
178	174	A48R	158	A49R	E	98.53	Thymidylate kinase	
179	175	A49R			E		Activating Wnt signaling by targeting the E3 ligase β-TrCP, a member of the Bcl-2 family, inhibiting NF-κB activation and promoting immune evasion and virulence	(266–268)
180	176	A50R	159	A50R	E	98.19	ATP-dependent DNA ligase	(269, 270)
181	177	A51R	160	A51R	E	95.51	Stabilizing microtubules, negatively regulating microtubule-dependent transport, and antagonizing cell-intrinsic antiviral response	(271, 272)
182	178	A52R			E		Inhibiting TLR-induced NF-κB activation, enhancing TLR-induced IL-1 production; host defense modulator	(263, 273)
183	179	A53R			I		Gene fragment, unknown function	
184	180	A55R	161	B1R	E	92.37	BTB kelch-domain containing protein, affecting virus-induced cytopathic effect and the outcome of infection; targeting importin-dependent NF-κB activation and inhibiting CD8 +T cell memory	(274, 275)
185	181	A56R	162	B2R	E	92.70	Enveloped virion envelope and cell membrane glycoprotein, hemagglutinin, inhibits cell fusion, complexing with K2 to form fusion regulatory proteins	(59, 60)
	181.5	No ortholog			E		Unknown function	
186	182	A57R			E		Guanylate kinase homolog	
187	183	B1R	163	B3R	E	97.32	Ser/Thr kinase, essential for viral DNA replication	(276–278)
188 a	184	B2R	164	B4R	E	92.06	Poxin, nuclease, cleaving cGAMP and restricting STING-dependent signaling	(279)
188	185	B3R	164	B4R	E	84.62	Schlafen-like, unknown function	
189	186	B4R	165	B5R	I	93.42	Ankyrin-like, possible role in virus spread	(280)
190	187	B5R	166	B6R	E	96.53	Enveloped virion type-1 membrane glycoprotein, located both on the membranes of infected cells and the enveloped virion envelope, required for trans-Golgi/endosomal membrane-wrapping of mature virion, affecting glycosylation, localization, and stability of A34 protein	(281–284)
191	188	B6R	167	B7R	E	86.31	Ankyrin-like protein, unknown function	
192	189	B7R	168	B8R	L	97.80	Virulence factor, located at the ER	(285)
193	190	B8R	169	B9R	E	95.13	Inhibiting binding of IFN-γ to receptor, host defense modulator	(286, 287)
195	191	B9R	170	B10R	I	95.65	Intracellular viral protein, unknown function	(288)
196	192	B10R			I		Unknown function	
197	193	B11R	171		E	90.28	Unknown function	
198	194	B12R	172	B11R	E	96.47	Ser/Thr pseudokinase, repressing viral DNA replication via a pathway antagonized by its paralog kinase B1	(289–291)
199	195	B13R	173	B12R	E	94.19	SPI-2 Serpin, anti-apoptosis	(292)
200	196	B15R	174	B13R	E	93.96	Soluble IL-1β receptor, binding IKK complex to inhibit IκBα phosphorylation and degradation to inhibit NF-kB	(293, 294)
201	197	B16R	175	B14R	I	87.57	Truncated IL-1β binding protein	
202	198	B17L	176	B15L	E	92.31	Unknown function	
203	199	B18R			E		Ankyrin-like, type I IFN binding protein	(295)
204	200	B19R	177	B16R	E	93.73	Unknown function	
205	201	Pseudogene (R)	178.5	B16.5R^*g*^	E	98.21	Unknown function	
205	202	B20R	178	B17R	E	86.96	Gene fragment, ankyrin-like protein, unknown function	
205	203		178	B17R	E	93.11	Gene fragment, ankyrin-like protein, unknown function	
206	204	No ortholog (R)			E		Kelch-like fragment, unknown function	
207	204.5	No ortholog (L)			I		Unknown function	
208	205	C12L	180	B19R	E	96.36	SPI-1 serpin 1,2,3 (Cop-K2L), apoptosis inhibition, inhibiting mouse IL-18 and promoting virus virulence, host defense modulator	(292)
209	206	C13L/C14L	181	B20R	I	95.79	Unknown function	

^*a*^ The nomenclature of the orthopoxvirus genes (OPG) is based on reference 296.

^*b*^ VACV-COP ORFs follow a systematic naming convention where ORFs are labeled starting with a letter based on the sizes of HindIII digestion of the genome (the largest fragment as A, the second largest as B, and so on), followed by a number indicating their order within each fragment block and R or L to indicate the right and left directions of the ORFs, respectively. The same naming convention is also used to name MPXV ORFs, which resulted in different or same names of the orthologs due to different sequences.

^*c*^ Temporal expression based on the earliest onset of expression. Some genes are expressed at multiple stages which are yet to be fully determined. E: early; I: intermediate; L: late; NE: not expressed.

^*d*^ The priority order for reference citations is relevance and then time of publication (oldest to newest).

^*e*^ This ORF secreted complement binding [C3b I isolates, but not in MPXV clade II (296).

^*f*^ D15L, D16L，and D17L are presented in some clade I isolates (296).

^*g*^ Newly annotated MPXV orthologs to VACV that were not previously annotated.

**Fig 1:**
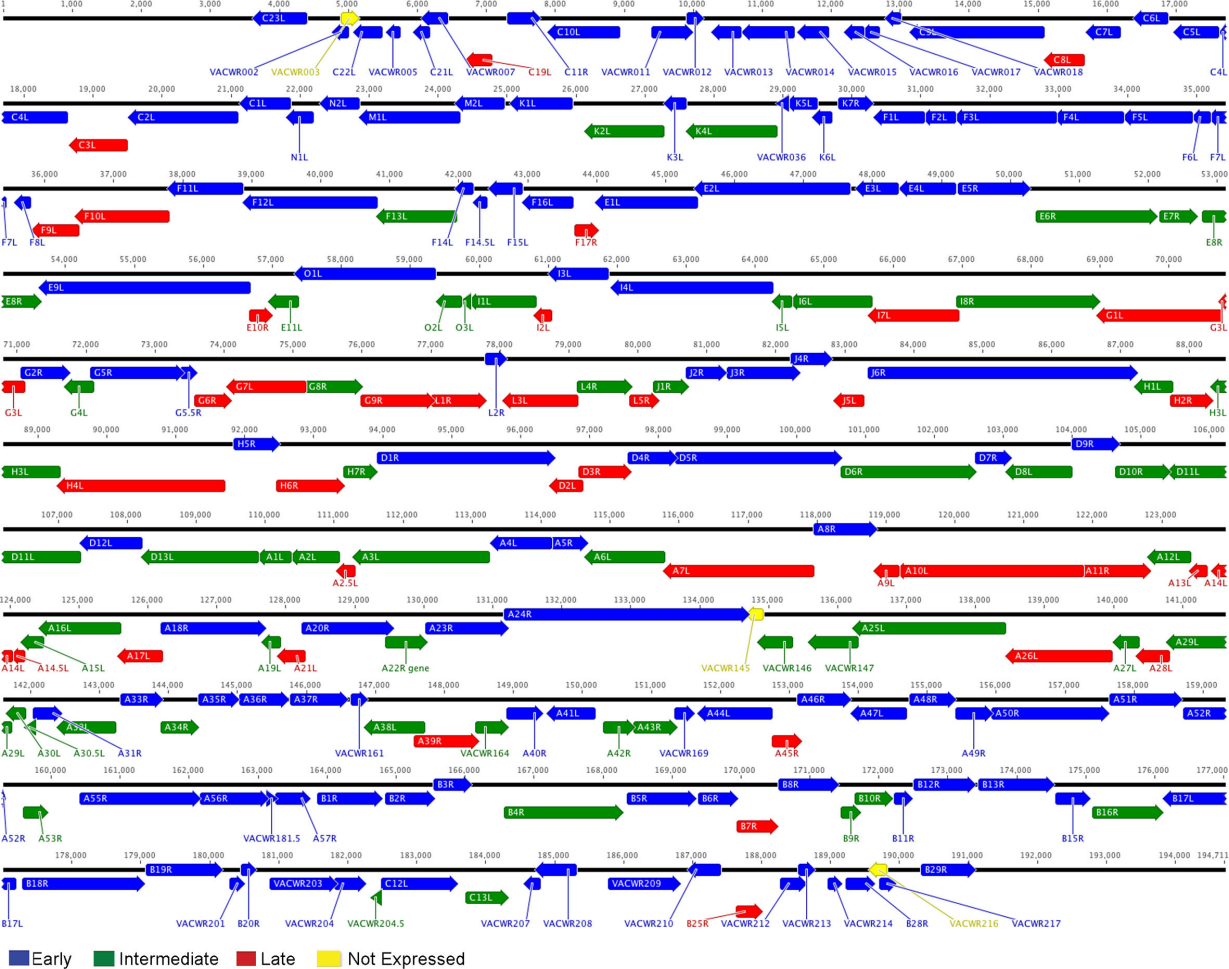
The transcriptome map of the VACV (WR strain) displays ORFs as colored arrows, indicating their transcription direction. When possible, the common HindIII fragment letter/number name from the Copenhagen strain is used to identify ORFs; otherwise, the VACV WR name is provided. The nucleotide positions on the VACV genome are numbered from 1 to 194,711. Each ORF is assigned the stage at which its earliest expression is detectable, though additional promoter elements that might contribute to later stages of gene expression are not shown. To be consistent with the mostly often used names in literature, the ORFs are mostly labeled with VACV-COP strain names, with some without VACV-COP orthologs labeled with VACV-WR names. The two ORFs (yellow) without RNA expression in RNA-seq were determined as not expressed. (Reprinted from reference 8.)

**Fig 2:**
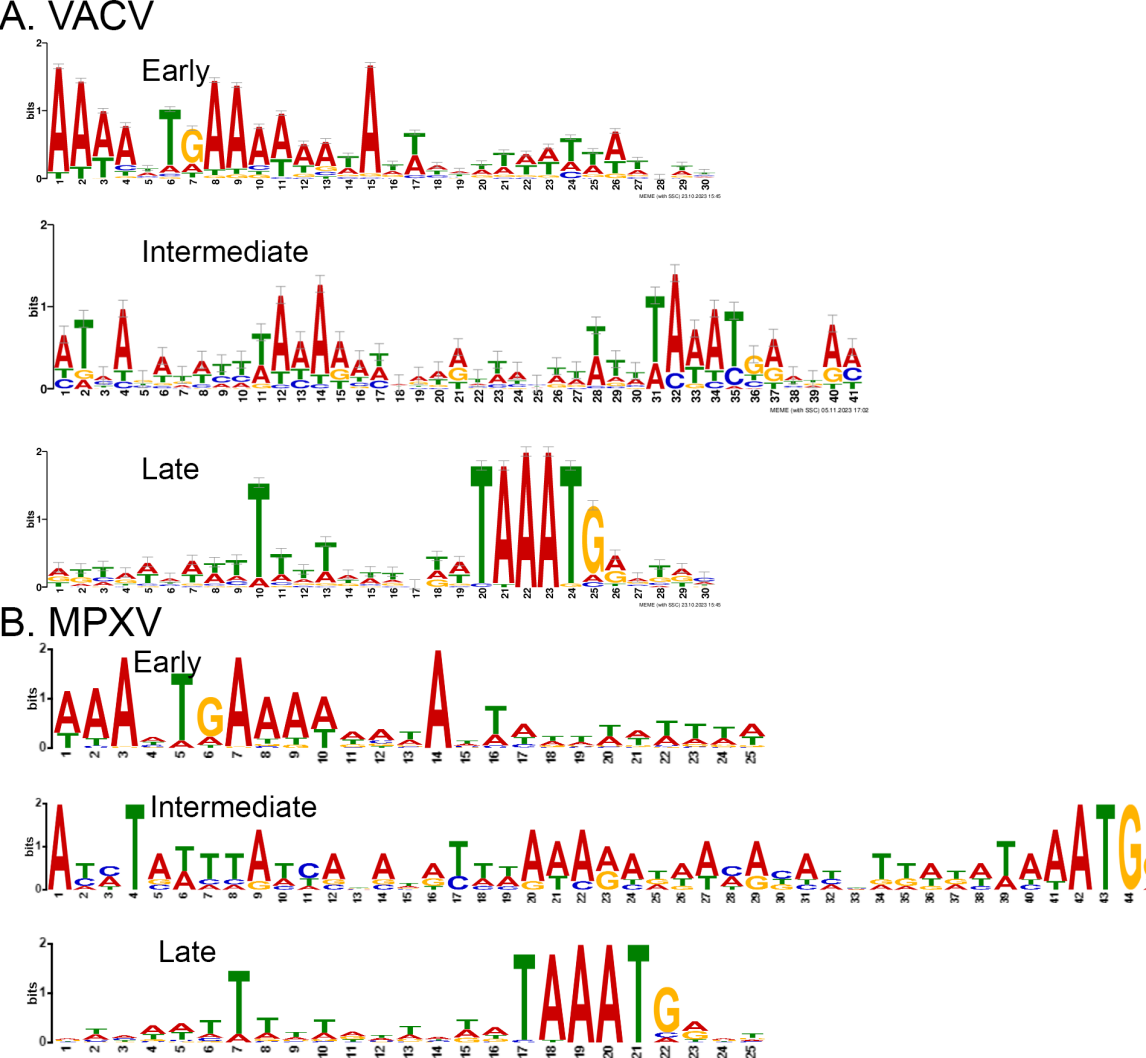
Motif logos of VACV (**A**) and MPXV (**B**) promoters were created using the MEME program by analyzing sequences that span 50 nucleotides upstream and five nucleotides downstream of the start codons (ATG). The numbers of the *X*-axis represent the positions of the conserved motifs of the promoters. The different lengths of the early, intermediate, and late promoter motifs are due to different lengths of the consensus sequences in each class of the promoter generated by the MEME program.

The classifications allowed the construction of a complete VACV WR strain temporal expression map (Fig. 1). The early genes are more clustered toward the genome’s two termini, while the intermediate and late ORFs are primarily distributed throughout the central region of the genome. Together with the work that precisely determined the transcriptional start sites of all early, intermediate, and late genes (297, 298), the consensus sequences of the early, intermediate, and late promoters were refined (Fig. 2A) (7–9). The early promoters are A-rich interrupted by TG. The late promoters contain a conserved TAAAT motif, typically followed by a G that overlaps with the start codons. Additionally, these late promoters feature a conserved T at the 10th nucleotide upstream of the TAAAT motif, surrounded by several less-conserved T residues. The intermediate promoters contain a TAAA sequence upstream of the start codons. However, only some of them followed with TG. The intermediate promoters also lack the T at the 10th nucleotide upstream of TAAA but feature a higher AT-rich sequence 15 to 19 nucleotides upstream, followed by a predominant T.

Correlation analyses of the temporal expression and gene functions/immune responses were also carried out in the previous studies (8). Genes in the three classes play distinct, kinetically coordinated roles in the VACV life cycle. Early genes predominantly function in DNA replication, transcription, host interactions, and the formation of the enveloped virion membrane. Intermediate genes are mainly involved in DNA-binding/packaging proteins and core-associated non-enzymatic proteins. Late genes primarily function in redox disulfide bond enzymes, morphogenesis-related proteins (such as crescent formation proteins), and mature virion membrane proteins, including components of the entry–fusion complex. This suggests a model of poxvirus replication: besides the transcription factors, early proteins create a virus-friendly cellular environment by interacting with host functions and preparing for DNA replication; intermediate proteins interact with the newly synthesized viral DNA genome for core morphogenesis; late proteins are more involved in the formation of the mature virion membrane and overall morphogenesis.

VACV proteins can be recognized as antigens by CD8+ T cells, CD4+ T cells, and B cells. Moutaftsi summarized the frequency of each VACV protein serving as antigens for these immune cells in individuals (299). Analysis of the distribution of top viral antigens across different classes revealed that the most immunodominant CD8+ T cell antigens are predominantly found in the early class, corroborating earlier notions (300, 301). In contrast, the top CD4+ T cell and B cell antigens are more prevalent in the intermediate class, suggesting that the kinetics of viral gene expression may influence immune specificity. This information has significant implications for vaccine design. For example, incorporating an early promoter could enhance the CD8+ T cell response.

## CLASSIFICATION OF TEMPORAL TRANSCRIPTION CLASSES OF MPXV GENES

MPXV was first identified in 1958 from monkeys (302). The first human case was reported in 1970. Sporadic outbreaks have occurred mainly in Central and West Africa (303). The number of mpox cases has increased rapidly since the 2010 s (304). In the past 2 years, MPXV has emerged in traditionally non-endemic regions, causing global outbreaks (305, 306), triggering two declarations of mpox as a public health emergency of international concern by the World Health Organization.

MPXV exhibited over 95% genetic similarity to VACV, with greater variation observed in the same terminal regions of the DNA. As the interest in understanding MPXV increases tremendously, we identified the orthologous genes in MPXV to those of VACV and assign their temporal expression classes with the assumption that the orthologs in MPXV and VACV are expressed in the temporal classes. This classification uses the MPXV Clade II isolate MPXV_USA_2022_MA001 genome (GenBank: ON563414.3) and VACV genome (NCBI Reference Sequence: NC_006998.1). Additional ORFs that are encoded in MPXV Clade I but not in Clade II are noted (Tables 1 and 2). The comparison of MPXV and VACV revealed an overall nucleotide identity of 97%, indicating the validation of this approach. To compare individual MPXV and VACV genes, we retrieved coding sequences (CDS) of the respective genes and aligned them based on their functions. The results showed that among the 186 MPXV genes analyzed, 178 have orthologs to VACV genes (Table 1). By analyzing nucleotide similarity between MPXV and VACV genes, we categorized orthologous genes based on sequence similarity percentages. Notably, some MPXV genes, such as MPXV J3, exhibit significant structural and compositional differences compared to their VACV counterparts, with MPXV J3 being unusually long and aligning with four separate VACV ORFs (VACWR005–008) in the VCAV WR strain, due to gene fragmentation.

**TABLE 2:** MPXV genes without orthologs in VACV

MPXV gene (based on MPXV-USA_2022_MA001)		Predicted function	Promoter motif	Predicted expression stage
004/187	D1L/N4R	Ankyrin	AATTATAAAAAATGAAAATCAA	E
005	D2L	Virion core component, morphogenesis	GATCTTATAGATAGATGTATTA	E
179	B18R	Kelch-like protein	ATATCGAAAATAATATACGTAA	E
182	B21R	Surface glycoprotein cadherin-like domain putative membrane-associated glycoprotein	TAATATGAAAAAAAACATAACT	E
183	B22R	N-methyl-D-aspartate (NMDA) receptor-like protein, R1R	AAAATGGAAATTAAAGCCCTC	E
184	N1R	Predicted to be involved in evading the host’s innate immune response	AAAATGGAAATTAAAGCCCTC	E
185	N2R	Unknown	AGATTATATATCGTTAAAAATC	E and/or I
186	N3R	NKG2D ligand OMCP, histocompatibility complex class I–like protein	TAGACCTATGAAATAAAAAAAG	E and/or I

In addition, we compared the nucleotides of individual VACV- and MPXV-likely promoters (from the 50th nucleotides upstream to five nucleotides downstream of start codon ATG) of the orthologs. Analysis revealed that most MPXV gene promoters demonstrated over 90% identity with their VACV counterparts. The majority of the remaining promoters, which showed lower identity, were linked to genes situated in the genome’s 5' and 3' regions, suggesting a relatively higher degree of flexibility in transcription regulation for these segments. Additionally, some promoters associated with pseudogenes may not have aligned precisely within the searched sequence, potentially introducing minor bias in the identity calculations. Upon closer examination of these lower-identity MPXV promoters, we concluded that their temporal expression likely aligns with that of VACV promoters. To further substantiate this, comparisons of amino acid sequences were conducted. The results showed that 156 of the 178 sequences shared more than 90% identity, reinforcing the presence of orthologous gene pairs between MPXV and VACV (Table 1). We then predicted and classified the 178 MPXV genes into early, intermediate, and late stages. The results showed that among the 178 MPXV genes, 96 are at the early stage, 47 are at the intermediate stage, and 36 are at the late stage; one is not expressed.

Interestingly, there are three ORFs in MPXV that were not annotated in the GenBank with a conserved ortholog in VACV, mostly small ORFs that likely were arbitrarily eliminated by the program’s ORF length cutoff. We added these MPXV ORFs in Table 1, including Q3L (35 aa), A39.5R (59 aa), and B16.5R (56 aa).

We then used the MEME (https://meme-suite.org/meme/tools/meme) to generate the conserved motifs of MPXV early, intermediate, and late promoters (Fig. 2B). The results show similar consensus sequences to VACV with minor variations and distinct patterns in the promoters at each stage. In the early stage, most promoters contained a TG sequence in the middle, with the rest of the sequences being A-rich. The late promoters had conserved signatures with TAAATG located at the start codons of the ORFs. Upstream of TAAATG is AT-rich with a preference for T. Intermediate promoters exhibited greater variability, with the majority containing a conserved TAAA sequence. Upstream of TAAA is AT-rich with a preference for A.

There are eight MPXV genes without orthologs in VACV. We predicted their transcription stages based on their promoter features by comparing them with the MPXV consensus promoter sequences and listed the predictions in Table 2.

## FUNCTIONAL ANNOTATIONS OF VACV GENES

To update the most recent functions of individual genes, we started by gathering the gene names, synonyms, and sequence information. PubMed (https://pubmed.ncbi.nlm.nih.gov) and Google Scholar (https://scholar.google.com) were searched for functions of individual genes. We then manually investigated and annotated all the gene functions. The functional annotation with the up-to-date literature reinforces the notion that early proteins modulate the cellular environment by interacting with host functions and preparing for DNA replication, while intermediate proteins are mainly involved in core morphogenesis, and late proteins participate in the formation of the mature virion membrane and morphogenesis to build fully infectious virions. Of note, while the previous studies have elucidated a great deal of information on many of the VACV gene functions in viral replication, the in-depth mechanisms of action of many of these genes are yet to be explored. Another critical area to understand the functions of the viral genes is how they functionally interplay with each other and host genes. The answers to these questions are key to understanding the complex poxvirus infection, transmission, and pathogenicity, as well as to developing poxvirus medical countermeasures and improving poxvirus-based vaccine vectors and oncolytic agents.

## SUMMARY

We summarized the temporal expression classification of the early, intermediate, and late genes VACV. Based on the orthologs in VACV and the consensus promoter sequences, we also assigned the temporal gene expression of MPXV. In addition, we annotated the functions of each of the genes. This review provides a valuable resource for researchers working on poxvirus biology, poxvirus utilities, and medical countermeasures.
